# Iron metabolism and ferroptosis in diabetic bone loss: from mechanism to therapy

**DOI:** 10.3389/fnut.2023.1178573

**Published:** 2023-05-05

**Authors:** Jiahao Bao, Yixuan Yan, Daihui Zuo, Zhiyong Zhuo, Tianhao Sun, Hongli Lin, Zheshen Han, Zhiyang Zhao, Hongbo Yu

**Affiliations:** ^1^Department of Oral & Cranio-maxillofacial Surgery, Shanghai Ninth People’s Hospital, College of Stomatology, Shanghai Jiao Tong University School of Medicine, National Center for Stomatology, National Clinical Research Center for Oral Diseases, Shanghai Key Laboratory of Stomatology & Shanghai Research Institute of Stomatology, Shanghai, China; ^2^Guangdong Provincial Key Laboratory of Stomatology, Hospital of Stomatology, Guanghua School of Stomatology, Sun Yat-Sen University, Guangzhou, China; ^3^Zhongshan School of Medicine, Sun Yat-Sen University, Guangzhou, China; ^4^Shenzhen Key Laboratory for Innovative Technology in Orthopaedic Trauma, Guangdong Engineering Technology Research Center for Orthopaedic Trauma Repair, Department of Orthopaedics and Traumatology, The University of Hong Kong-Shenzhen Hospital, Shenzhen, China; ^5^School of Public Health, The University of Hong Kong, Pok Fu Lam, Hong Kong SAR, China

**Keywords:** osteoporosis, bone, diabetes, iron metabolism, ferroptosis, mechanism

## Abstract

Osteoporosis, one of the most serious and common complications of diabetes, has affected the quality of life of a large number of people in recent years. Although there are many studies on the mechanism of diabetic osteoporosis, the information is still limited and there is no consensus. Recently, researchers have proven that osteoporosis induced by diabetes mellitus may be connected to an abnormal iron metabolism and ferroptosis inside cells under high glucose situations. However, there are no comprehensive reviews reported. Understanding these mechanisms has important implications for the development and treatment of diabetic osteoporosis. Therefore, this review elaborates on the changes in bones under high glucose conditions, the consequences of an elevated glucose microenvironment on the associated cells, the impact of high glucose conditions on the iron metabolism of the associated cells, and the signaling pathways of the cells that may contribute to diabetic bone loss in the presence of an abnormal iron metabolism. Lastly, we also elucidate and discuss the therapeutic targets of diabetic bone loss with relevant medications which provides some inspiration for its cure.

## 1. Introduction

Osteoporosis, which is regarded as the most common bone illness worldwide, has the characteristics of low bone mass, bone tissue’s microarchitectural deterioration, and declined bone strength (1). It has been determined that diabetes-specific bone characteristics, such as deficiencies in glucose/insulin metabolism, the buildup of advanced glycosylated end products (AGEs), and a lack of bone microvasculature, may constitute a novel syndrome that can be categorized as diabetic osteoporosis (DOP) (2). DOP has eclipsed other diabetes-related illnesses as the major cause of death and mutilation, substantially affecting patients’ quality of life and inflicting a substantial financial burden on their families and society (3–6). Current glucose-lowering therapeutic measures mainly consist of metformin and sulfonylureas. However, their efficacy might be enhanced. Meanwhile, a few therapies targeting diabetic mellitus were discovered to increase the possibility of fractures, such as Thiazolidinediones (TZDs) and possibly sodium-glucose cotransporter-2 (SGLT2) (2, 7, 8). Investigating the mechanisms underlying DOP can contribute to the development of new therapeutic strategies, despite the fact that researchers do not fully comprehend these mechanisms. Recent studies have demonstrated that the onset of Type 2 Diabetic Osteoporosis (T2DOP) may be correlated with the buildup of peroxides and reactive oxygen species (ROS) resulting from ferroptosis. It is also investigated that some signal molecules and signal pathways, such as NRF2/HO-1/GPX4 and SLC7A11 can ameliorate the above symptoms, providing novel possible therapeutic targets and research directions for T2DOP (9–11).

Iron metabolism is the process of iron being absorbed, transported, distributed, stored, utilized, transformed, and excreted in living organisms. The metabolism of iron is of great significance for cells. It has been discovered that iron can switch between its ferric (Fe^3+^) and ferrous (Fe^2+^) forms, allowing it to take and give electrons with relative ease (12). Therefore, iron metabolism is crucial to the regular functioning of several intracellular processes, and the disturbance of iron homeostasis could potentially increase the risks of many diseases. For example, iron deficiency is perceived as one of the most prevalent causes that induces anemia, while iron overload is recognized as one of the main culprits of heart diseases, bone diseases, and cognitive level-related diseases (13–17). As a significant mechanism for disease exploration, iron metabolism has gotten attention from plenty of research in exploring the relationship between iron metabolism and bone metabolism and the underlying pathways that induce osteoporosis (18–20).

Iron metabolism also impacts bone homeostasis through ferroptosis, which is a kind of iron-dependent cell death characterized by an aggregation of lipid peroxides and ROS (21). Ferroptosis has been found to be associated with the pathophysiology of diverse ailments, which includes malignant tumors, ischemic diseases, neurodegenerative diseases, as well as metabolic disorders. Substances that are induced by ferroptosis performs the ability to diminish the activated state of glutathione peroxidase 4 (GPX4) via multiple routes, resulting in a significant decrement in oxidation resistance and oxidative death in cells eventually. ROS buildup has a significant impact on the creation and survival of osteoblastic cells and their differentiation into osteocytes, thus, oxidative stress might be a major contributor to T2DOP. Wang et al. discovered ferroptosis in T2DOP rats’ bone tissue, and therapies with ferroptosis inhibitors dramatically could reduce the stress of oxidation and ameliorates the symptoms of osteoporosis, although the underlying mechanisms were still far from fully understood (19).

Furthermore, findings from existed publications been proved that some medications that targeting on iron metabolism, including melatonin, Qing’e pills (22), and Artesunate (ART) (23) could relieve the systems of T2DOP to some extent, indicating the necessary to deeply invest in the research of iron and bone metabolism. Therefore, this review intends to systematically summarize the research progress of iron metabolism and diabetic bone loss, the underlying mechanisms exploration, and clinical therapies for the comprehensive evidence of further study.

## 2. Bone fragility in diabetes

According to the International Diabetes Federation, the alarming number of diabetics worldwide has surpassed 537 million as of 2021, and the most striking features of diabetes include chronically higher-than-standard fasting and random blood glucose, which either induce insulin deficiency [Type 1 diabetes mellitus (T1DM)] due to damage to pancreatic beta cells or progressive insulin secretion defect [Type 2 diabetes mellitus (T2DM)] from insulin resistance (24).

Diabetic complications could significantly increase the patient’s risk of morbidity and mortality. Long-term diabetes is known to cause macrovascular and microvascular damage to the heart, brain, nerves, eyes, and kidneys, while significantly less attention has been given to the musculoskeletal system. The high glucose (25) environment brought on by these two factors could further potentially affect the bone metabolism, bone loss or even osteoporosis (26). Osteoporosis is defined as bone mineral density (BMD) at the femoral neck that is 2.5 standard deviations (SD) or more below the mean for young female adults (T-score less than or equal to −2.5 SD (27)), on the basis of dual-energy X-ray absorptiometry (DXA). Osteoporosis induced by diabetes mellitus, sometimes referred to as diabetic bone disease, is a chronic disease that subsequently increases bone fragility and fracture risk owing to a decrease in bone density and damage to the bone microstructure (28, 29). Research found that patients with diabetic bone disease are at a higher risk of long-term bone pain, motor dysfunction and fractures (30). More than 35% of individuals with Type 2 diabetes displayed bone loss, with 20% meeting the diagnostic criteria for osteoporosis.

Diabetic bone loss is characterized by altered bone density, altered bone turnover, reduced bone microarchitecture, and increased fracture risk. Multiple independent research demonstrate that the BMD of diabetic individuals may be decreased, constant, or even enhanced. The femur and vertebrae are the major sites of elevated BMD in patient with T2DM (31–33). Generally, having unnecessarily abundant energy and being overweight are the main causes of the rise in BMD in T2DM patients. Adaptive changes in the bone that enable the body to sustain a heavier load may also contribute to the increment of BMD (34, 35). Nonetheless, despite greater mean BMD and T-score values, there is increasing evidence that the T2DM-associated increased fracture risk is related to decreased bone quality, which may be termed “diabetic osteopathy” (36–38). The apparently contradictory finding is based on the changes in bone turnover, decreased bone microarchitecture, accumulation of AGEs, muscular weakness, anti-diabetic medication, etc., which might have the possibility to enhance the fracture risk of T2DM patients (39). Patients with T2DM usually display aberrant bone microstructure, particularly in the cancellous bone, with both a reduction in the number of trabeculae and morphological defects (40); also they also have a considerably decreased number of trabeculae and trabecular thickness in the femoral head compared to non-diabetic patients (41). Thinner cortical bones and higher porosity show a direct correlation with a decreased breaking load. Compared to the general population, individuals with T2DM had a 3% drop in radial cortical bone density and a 25% increase in cortical bone porosity (42); a smaller cross-sectional area, more cortical porosity; and a lower cortical vertebral BMD in the tibia, but not the radius with the assistance of HR-pQCT (43).

## 3. Bone cell biology in high glucose condition

**Figure 1:**
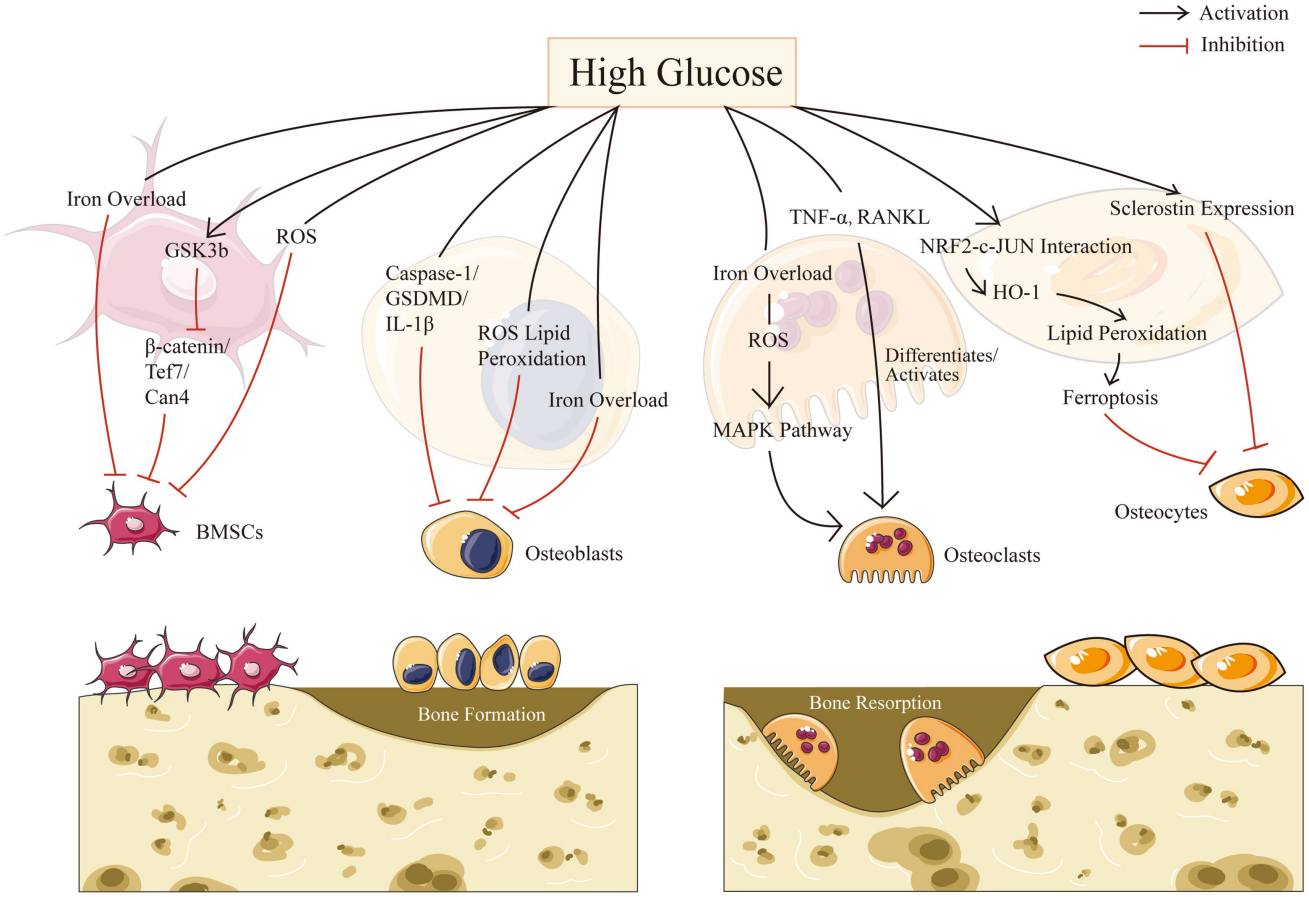
Bone cell biology in high glucose condition.

Resorption and creation of bone are two essential components of bone remodeling. A major element in the development of osteoporosis is an imbalance in bone reconstruction. Bone remodeling, the coordinated activities of bone-resorbing osteoclasts and bone-forming osteoblasts, is required for continuous bone turnover and regeneration. Diabetes may affect all types of bone cells and promote adipose tissue formation in bone marrow. In this part, we intend to describe separately for four different cells in bone microenvironment in the context of HG: mesenchymal stem cells, osteoblasts, osteoclasts, and osteocytes (Figure 1).

### 3.1. Mesenchymal stem cells in HG condition

Osteoblasts are derived from multipotent mesenchymal stem cells (MSCs), which may move to the site of impairment, proliferate, and differentiate (44). MSCs may be separated from peripheral blood and nonhematopoietic tissues such as adipose tissue, trabecular bone, dermis, dental pulp, synovium and lung, despite the fact that bone marrow is assumed to be the primary source of these precursor cells (45). As the most significant MSCs obtained from bone marrow, bone marrow-derived mesenchymal stem cells (BMSCs) play crucial roles in bone tissue regeneration. Different microenvironments such as high glucose levels, inflammation, and hypoxia, would change the physiological functioning of stem cells (46). Recent study has shown that osteoporosis was associated with an increase in circulating MSCs with low osteogenic potential, highlighting the importance of BMSCs for successful bone remodeling and/or repair *in vitro* (47). A number of studies have shown that the biological activities of BMSCs were modified by chronic exposure to a diabetic pathogenic environment (48, 49).

In addition, the serine/threonine kinase glycogen synthase kinase-3, also known as GSK-3, contains two remarkably homogeneous isoforms, GSK-3a and GSK-3b, which is a broadly expressed enzyme (50). GSK-3b inhibition could increase bone density (51). In high glucose microenvironments, GSK-3b activation as well as Wnt pathway suppression impede BMSC migration and proliferation, however, lithium chloride, an inhibitor of GSK-3b, may restore the functionality of BMSCs (46), according to Zhang et al. Moreover, Yu’s study demonstrated the activation of GSK3b in diabetic osteoporosis and its deleterious osteogenic affected BMSCs in a high glucose milieu through the β-catenin/Tcf7/Ccn4 signaling axis inhibition, and thus provide unprecedented perspectives into diabetes osteopathy (48).

Furthermore, as a common denominator of the numerous osteogenic signaling pathways, it’s suggested to strictly mange the ROS levels for MSCs to undergo osteogenic differentiation (52). It is reported that usage of deferoxamine *in vitro*, the anti-osteogenic impact of superparamagnetic iron oxide nanoparticles was abolished, indicating that the free form of iron is significant to the inhibition of MSCs from differentiating into osteoblasts (53). Balogh et al. also approved that iron specifically prevents BMSCs from differentiating into osteoblasts without affecting adipogenic or chondrogenic differentiation (54).

In summary, high glucose condition shows an impact on mesenchymal stem cells and suppresses its differentiation process.

### 3.2. Osteoblasts in HG condition

Osteoblasts, which serve as bone-forming cells, originate from the sequential activity of transcriptional factors on mesenchymal precursors to osteoprogenitor lineages and eventually differentiate into osteocytes. Osteoblasts produce extracellular proteins such as osteocalcin, alkaline phosphatase, and type I collagen, the latter of which accounts for more than 90% of bone matrix protein. The extracellular matrix is initially secreted as unmineralized osteoid and becomes gradually mineralized when calcium phosphate concentrates as hydroxyapatite (55).

It has been demonstrated that the high glucose conditions in T2DM severely impair the biological functions of osteoblasts, resulting in an increase in the density of mitochondrial bilayers and a decrease in the number of mitochondrial cristae, and leading to the accumulation of ROS as well as lipid peroxides causing the cells to exhibit excessive oxidative stress as well as lipid peroxidation, and causing the cells to exhibit excessive oxidative stress and lipid peroxidation, accelerating apoptosis and autophagy of osteoblasts. It is reported that the proliferation and differentiation of osteoblasts could be inhibited by excessive glucose in alveolar bone through the caspase-1/GSDMD/IL-1 pathway, indicating the opposite effects from usage of caspase-1 inhibitors *in vivo* and *in vitro* (56).

HG condition could also affect osteoblasts by modulating iron metabolism as well. It was identified that iron overload reduces MC3T3 cell viability and causes apoptosis, in which they reported that an excess of iron may partially suppress osteoblast activity, and disturb the differentiation and mineralization processes of osteoblasts (57). What’s more, the pathogenesis of T2DOP was significantly influenced by the osteogenic activity of osteoblasts, which was negatively influenced by iron overload caused by the increased expression of DMT1 in osteoblasts (58, 59).

In summary, HG condition not only suppress the differentiation process of osteoblasts, but also strongly affected its osteogenic function.

### 3.3. Osteoclasts in HG condition

Osteoclasts are end-differentiated multinucleated cells of the monocyte/macrophage lineage with unique function of resorbing bone matrix (60). Osteoclasts break down bone by secreting acids and proteolytic enzymes such cathepsin K, also known as CTSK, which break down matrix components like collagen during osteoclastogenesis (61, 62). As was known, monocytes could only differentiate into osteoclasts *in vitro* when co-cultured with cells comprising stromal cells and osteoblasts (63). Because osteoclasts and osteoblasts’ respective bone resorbing and building processes are closely correlated, an adult’s bone mass is generally steady. However, in many disease states such as osteoporosis, metastatic bone cancer, and inflammatory arthritis, the delicate balance is disturbed by an increase in osteoclast bone resorption activity (60).

Various studies have revealed that the high glucose condition has a certain promotion effect on the differentiation of osteoclasts, which can strengthen their bone resorption ability (64, 65). Clinical studies showed that osteoclastogenesis was more frequently accelerated by diabetes mellitus: (a) enhanced levels of tartrate-resistant acid phosphatase, a sign of increased osteoclast activity, were found in the blood of patients with T2DM (66); (b) tartrate-resistant acid phosphatase levels were higher in blood among T2DM patients (67). Studies on animals’ models further approved that diabetes patients have higher osteoclast activity (68, 69): compared to normoglycemic controls, osteoclastic bone resorption was increased in T2DM rats (70). TNF-a, macrophage-colony stimulating factor, receptor activator of nuclear factor kappa-B ligand (RANKL), as well as the vascular endothelial growth factor-A were all elevated in diabetic mice, which would differentiate and activate osteoclasts (71–73).

Furthermore, osteoclasts could also be significantly influenced by iron overload, which is induced by DM. It’s reported that ROS that arises from iron overload could activate the MAPK pathway, improving the differentiation capability and the bone resorption capacity of osteoclasts in bone metabolism (20). There’s also evidence showed that ferritin autophagy took place when cells were iron-deficient, which makes them more susceptible to ferroptosis caused by intracellular Fe^2+^ (74, 75) Additionally, Mature osteoclasts require a greater amount of cytoplasmic free iron than other osteocytes. Hence, osteoclasts are more susceptible to ferroptosis (76, 77).

In summary, it implies that the HG condition can influence osteoclast activity, which may result in aberrant bone metabolism and osteoporosis.

### 3.4. Osteocytes in HG condition

Osteocytes are terminally developed osteoblasts that undergo substantial morphological changes when embedded in the mineralized bone matrix. It plays a key function throughout the homeostasis regulation of bone, with a main function to communicate with the surrounding environment (78, 79): (a) their numerous dendritic processes that protrude from the osteocyte soma in all directions and enter the ‘canaliculi’, which are tiny passageways by which the osteocytes could connect with other osteocytes and cells in the bone marrow or periosteum; (b) osteocytes in the interstitial tissue of the lacunar-canalicular structure come into touch with liquid, which enables these cells to function well. Consequently, the osteocyte lacunar-canalicular network provides a vast system that could detect changes in bone loading and regulate bone remodeling for the healthy skeleton, with the collaboration of other bone cells’ (osteoblasts and osteoclasts) activities (80).

Osteocytes may release various signaling substances in response to loading or unloading stimuli via the SOST/DKK/Wnt or the RANKL/Osteoprotegerin (OPG) axis. It may either promote bone resorption by producing RANKL and decreasing OPG, or decrease bone resorption by flipping the RANKL/OPG ratio. Osteocytes are also the substantial producers of Dkk1(the Lrp5/6 Wnt signaling inhibitor) and sclerostin (transcription product of the SOST genes) in connection to bone formation (78, 81, 82). It’s interesting to note that patients with T1DM and T2DM had higher serum levels of sclerostin (83, 84), indicating variations in glucose concentration may have impact on the cells most crucial for maintaining bone health as sclerostin is largely produced by osteocytes. Moreover, Blood glucose levels significantly above and below the normal range of 80–140 mg/dl may have detrimental effects on osteocytes (85). Another study showed that diabetes caused osteocytes to alter over time and upregulate the sclerostin gene, that might be mediated by local glucose concentrations and could have a significant effect on the deterioration of bone quality (85–87).

Furthermore, it’s suggested that inhibiting the ferroptosis pathway in diabetic mice prevented DOP and osteocyte death (10). Traditional cell death inhibitors such as Z-VAD-FMK and Nec-1 had no impact in rescuing osteocytes from the death induced by high glucose and high fat (HGHF) circumstances. Furthermore, they concluded excessive lipid peroxidation may be the primary source of cell damage in the diabetic milieu and that ferroptosis may be strongly associated with the underlying molecular process of cell osteocyte death. Altogether, high glucose level could induce longstanding changes in osteocytes via upgrading sclerostin expression and inducing ferroptosis, resulting in the imbalance of bone metabolism eventually (10).

In summary, the high glucose level in the blood caused by T2DM alters the dynamic equilibrium between bone formation and bone resorption in a normal organism, resulting to a variety of complications such as T2DOP.

## 4. Iron-related protein and bone formation in HG

Studies have approved that proteins involved in iron metabolism have a very clear connection to bone metabolism. Here we give some summaries. Table 1 summarizes iron-related proteins and bone metabolism in high glucose condition.

**Table 1:** Iron-related proteins and bone metabolism in high glucose condition.

Protein	Mechanism	Effect in Ferroptosis	Basic foundation	Origin and distribution *in vivo*	Biochemistry and molecular structure	References
Ferritin	ROS/PINK/Parkin	Ferroptosis in OB, osteocalcin and CBF-a1 inhibition	Primary iron storage proteins of most living organisms, members of a broad superfamily of ferritin-like diiron-carboxylate proteins	Almost all body tissues especially liver cells and reticuloendothelial cells	Iron-free (apoferritin) molecule is a protein shell composed of 24 protein chains arranged in 432 symmetry. 2 types of chains (subunits): H or M (fast) and L (88), which differ in rates of iron uptake and mineralization.	(74, 75, 89)
HEP	BMP/SMAD	Increased ROS production, mitochondrial biogenesis, and PGC-1β expression in osteoclasts	An antibacterial and antifungal protein	Expressed in the liver	Cysteine-rich, forms a distorted beta-sheet with an unusual disulphide bond found at the turn of the hairpin.	(90–95)
Tfr	BMP/p38MAPK/Wnt	Tfr1 is a key player in the uptake of iron-loaded transferrin into cells, Tfr2 binds transferrin but with a significantly lower affinity than Tfr1	TfR1 may also participate in cell growth and proliferation	Tfr1: widely expressedTfr2: hepatocytes, hematopoietic cells, and duodenal crypt cells	Tfrs are homodimeric type II transmembrane proteins containing three distinct domains: protease-like, apical or protease-associated, and helical domains.	(96–104)
IRP	Through post-transcriptional regulation of iron metabolism-related proteins to maintain cellular iron homeostasis	Decreased expression of bone formation markers such as TFRC and ferritin	Sustaining normal mitochondrial function			(105–107)
METTL3	Upregulating the ASK1/p38 signaling pathway to induce ferroptosis	Induction of ferroptosis in OB	Regulating various processes such as the circadian clock, differentiation of embryonic and hematopoietic stem cells, cortical neurogenesis, response to DNA damage, differentiation of T-cells and primary miRNA processing, playing an important role in various kinds of tumors	Almost all body tissues		(108–111)
DMT1		Suppression of the OB osteogenic function	Having a role in gastrointestinal uptake of metals and in transferrin dependent trafficking of iron and manganese, Cu2+, Cd2+	Widely expressed	DMT1 is a 12-transmembrane-domain protein, having at least four isoforms: two are derived from N-terminal alternatives and two are from C-terminal alternatives	(112–115)
HO-1	NRF2 and c-Jun/HO-1	Catalyzing heme oxidation to produce a significant amount of free labile iron, inducing ferroptosis in osteocytes	Catalyzing heme degradationholds antioxidant, anti-inflammatory, cytoprotective, proliferative, and angiogenic properties	Expressed in low quantities under normal conditions except in tissues that involve the degradation of senescent red blood cells, such as the spleen, liver, and bone marrow		(89, 116–120)
GSH	XC-system/GSH/GPX4 axi	Reduced osteoblast ferroptosis and enhanced osteogenic activity.	Converting peroxide (R-OOH) into alcohol (R-OH) and decreasing the toxicity of lipid peroxides	Widely expressed		(19, 121, 122)

HEP, hepcidin; Tfr2, transferrin receptor 2; IRP2, iron regulatory protein 2; METTL3, methyltransferase-like 3; DMT1, divalent metal transporter 1; HO-1, heme oxygenase-1; GSH, glutathione; OB, osteoblast.

### 4.1. Ferritin

Zarjou et al. found that the ferroxidase activity of ferritin was responsible for the suppression of osteoblasts’ activities (75). By observing the effects of ceruloplasmin (a protein with ferroxidase activity but no iron sequestration ability) and examining the osteoblast-specific genes expression, they discovered that ferritin ferroxidase activity might inhibit the production and subsequent activity of alkaline phosphatase (ALP). Thus, the ferritin ferroxidase activity could not only inhibit the exclusive osteoblast product osteocalcin which in turn affect calcification, but also downregulate the osteoblast-specific genes such as core binding factor α-1, alkaline phosphatase and osteocalcin (75).

Additionally, it has been demonstrated that mitochondrial ferritin (FtMt) reduces oxidative stress and maintains intracellular iron homeostasis (25). If FtMt expresses excessively, it will lessen ferroptosis that happens in osteoblasts under HG environments, whereas if FtMt becomes silent, it can stimulate autophagy in mitochondrial via the ROS/PINK1/Parkin pathway, leading to an increase in osteoblasts ferroptosis (74). In T2DOP, FtMt was showed to prevent ferroptosis in osteoblasts by decreasing oxidative stress produced by excess ferrous ions, while FtMt deficiency increased mitophagy in the pathogenesis of T2DOP (74, 89).

### 4.2. HEP

Hepcidin (HEP), which is produced and secreted by liver cells, regulates iron homeostasis. It can connect to the ferroportin (FPN) receptor, which is a type of transmembrane protein, to prevent cellular iron from entering the bloodstream (11, 123). The sole iron output protein in vertebrates up to this point is FPN (90–92). If FPN activation induced by HEP is inadequate or inefficient, the organism may experience iron overload and perhaps iron deposition in the skeletons. Causing numerous ROS production, mitochondrial biogenesis, peroxisome proliferator-activated receptor gamma coactivator-1beta (PGC-1β) expression in osteoclasts and ultimately resulting in osteoporosis (93). In addition, there’s also a study concluding that BMP/SMAD signaling pathway was discovered to possess the ability to regulate the expression level of HEP (94). Xu et al. not only found that HEP stimulated osteoblast intracellular Ca2+ in a dose-dependent manner, but also revealed that the process mention above is facilitated by voltage-dependent L-type calcium channels, which indicated an unignorable effect that HEP had on bone metabolism (95).

### 4.3. Tfr2

In mammalian cells, there are two distinct transferrin receptors (Tfrs) (96). Transferrin receptor 1 (Tfr1) is predominantly expressed and binds to Fe^3+^-loaded holo-Tf with great affinity. Plasma iron flows attached to the iron transporter protein transferrin and is absorbed by endocytosis that mediated by Tfr1 under physiological circumstances. Tfr1 is regulated post-transcriptionally by intracellular iron status through the iron-regulatory protein system (97), resulting in elevated Tfr1 under low iron circumstances and diminished Tfr1 under high iron conditions (98). Bhaba’s reported that Tfr1absence resulted in a >50% drop in osteoclast lineage cells in the total osteoblasts intracellular iron concentration (99). However, Tfr1-deficiency had no impact on the iron levels in monocytes and pre-osteoclasts. It has been determined that mature osteoclasts procured extracellular iron mostly via using Tf and heme (99). This study found that iron uptake regulated by Tfr1 is a key iron acquisition route in osteoclast lineage cells, which significantly regulates bone remodeling of trabecular in the perpendicular and axial bones via female and male mice models (99). Also, the increased cytoplasmic iron generated by Tfr1 was approved to be especially essential for mitochondrial energy consumption and cytoskeletal structure in osteoclasts, however, it still showed slight impact on the differentiation of osteoclasts (99).

Transferrin receptor 2 (Tfr2) is another crucial regulator of hepcidin, which is proposed to control iron homeostasis. Tfr2 is known for controlling systemic iron levels, but it also promotes healthy erythropoiesis (100–103). Tfr2 has recently been identified a novel extrahepatic function, controlling bone mass directly by osteoblasts in the research from Martina Rauner’s team (104). They reported that Tfr2, which is predominantly located in osteoblasts, governed bone production but had little effect on the systemic iron homeostasis. Furthermore, Tfr2 could also activate p38 MAPK signaling in osteoblasts, which leads to the induction of the Wnt inhibitor sclerostin and limits bone formation, hence, Tfr2 functions as a unique regulator of bone mass via modifying the BMP-p38 MAPK-Wnt signaling axis (104).

### 4.4. IRP

Iron regulatory protein 1 (IRP1) and iron regulatory protein 2 (IRP2) post-transcriptionally control the metabolism of iron in vertebrate cells (105). Zhang et al. demonstrated that iron drove the transcription of NADPH oxidase 4 (NOX4) by dissociating IRP1 and thereby depressed osteogenesis in bone metabolism. Mechanically, they revealed that the NOX4 locus includes iron-response element-like sequences, which are bound by IRP1. Upon iron binding, IRP1 dissociates from the IRE-like sequences, resulting in the activation of NOX4 transcription. Osteoblasts with increased NOX4 accumulate lipid peroxide and show obvious alterations in mitochondrial morphology and function (106). In mouse bone tissue after the deletion of IRP2, investigation has discovered the expression of the genes for the proteins that served as iron transporter (FLT, FPN1, and TFR1). This is a disease characterized by scant trabecular bone, which could induce the reduction of iron concentration and the downregulated expression of bone formation markers (107). Therefore, a lack of IRP2 may prevent the iron transporter from transferring, which results in a lack of iron and affects bone metabolism. However, additional research will be required in the future to understand this conclusion because the underlying process is currently elusive.

### 4.5. METTL3

Methyltransferase-like 3 (METTL3), one of the m^6^A writers, is approved to play a role in the pathophysiology and growth of bone-related disorders including osteoporosis, arthritis, and osteosarcoma (108). Nonetheless, there is controversy regarding the link between osteoporosis and METTL3 expression. For instance, one study found that overexpression of METTL3 in bone marrow monocytes protected mice against osteoporosis induced by estrogen deprivation, while disruption of METTL3 in mice destroyed bone formation, decreased osteogenic differentiation, and improved marrow obesity (109). Another study demonstrated a negatively regulatory role of METTL3 in osteogenesis process by activating NF-κB pathway, which was considered as a significant osteogenic differentiation inhibitor. And METTL3 was found to induce the expression of MYD88, an upstream regulator of NF-κB pathway, through control m6A methylation status of MYD88-RNA (110).

Furthermore, researchers have discovered that METTL3 may be involved in high glucose and palmitic acid (HGPA)-induced osteoporosis via activating the ASK1/p38 signaling pathway, in which they noticed that METTL3 knockdown prevented HGPA-induced activation of ASK1/p38 signaling (111). The fact that the expression of the ferroptosis-inhibitory proteins GPX4 and SLC7A11 was markedly repressed further provided evidence that activating ASK1/p38 pathway was responsible for the induction of ferroptosis (111).

### 4.6. DMT1

Divalent metal transporter 1 (DMT1) is a 12-transmembrane-domain protein that is present in various tissues, such as bone, kidney, and duodenum. DMT1 transports lots of divalent cations (112). It is the main apical transporter in charge of absorbing intestinal Fe^2+^ and it is found to be widely expressed in endosomal compartments, in a place where it is in responsibility of exporting Fe^2+^ throughout the transferrin cycle (112, 113). As a result, iron overload and DMT1 expression are closely connected. DMT1 plays a role in the absorption of other metals in addition to its role in the metabolism of iron and manganese, and it also involves in the transfer of Cu^2+^ and Cd^2+^ (114, 115).

Further studies proved that the overexpression of DMT1 could lead to iron overload in osteoblasts, thus suppressing the osteogenic function of osteoblasts. Liu et al. discovered that human hFOB1.19 osteoblasts treated with ferric ammonium citrate (FAC) expressed more DMT1 compared with those untreated cells (58). Zhang et al. found that there were less of the autophagosome accumulation that was caused by FAC in DMT1-shRNA hFOB1.19 cells, which suggested that DMT1 controls the levels of Fe2+ in osteoblasts, which has an impact on the cellular accumulation of autophagosomes (59). In summary, DMT1 expression could enhance in the bone tissue of type 2 diabetic condition, then DMT1 induces iron overload in osteoblasts, and ultimately affects the osteogenic function of osteoblasts.

### 4.7. HO-1

Heme oxygenase-1 (HO-1) is a cellular inducible oxidative stress regulator that oxidizes heme to produce biliverdin, carbon monoxide, and free ferrous iron (116). The role HO-1 plays in ferroptosis is still up for dispute at this time. Numerous studies showed that elevated HO-1 expression prevented oxidative stress in cells and prevented ferroptosis (37, 117, 118). For instance, Adedoyin et al. discovered that HO-1^−/−^ cells demonstrated higher erastin-induced cell death when compared to HO-1^+/+^ renal proximal tubule cells (119). Other researchers, however, identified that excessive HO-1 caused organ failure and exacerbated ferroptosis (89, 120). According to Fang et al., inhibiting HO-1 expression reduced ferroptosis in cardiomyopathy in models *in vivo* and *in vitro* (89). Tang et al. noted that blocking HO-1 activity should be a reliable way to prevent ferroptosis in the retinal pigment epithelium (120). It therefore demonstrated that HO-1 was a double-edged sword that functions differently in distinct tissues and disease models.

HO-1 plays important roles in bone metabolism. Yang’s team approved that the group with DOP had much more lipid peroxidation occurred *in vivo* via DOP mouse model, indicating that the high-glucose microenvironment could induce osteocyte ferroptosis. Then they went further demonstrated the concrete mechanism of how high-glucose microenvironment induced intracellular iron overload. In diabetic microenvironment, HO-1 transcription was activated upstream by the heterodimer of NRF2 and c-JUN and activation of HO-1 catalyzes heme oxidation produced a significant amount of free labile iron (10). What’s more, Ma’s finding also supports the theory that HO-1 might mediate HGHF-induced osteocyte ferroptosis (9). HO-1 activation and ferroptosis are both mutually causal and can lead to an endless loop of mutual promotion (10, 121).

### 4.8. GSH

Ferroptosis can also be induced by the depletion of glutathione (GSH) and the reduction in GPX4 activity (121). GSH is a protective substance in cells and the main substrate of GPX4, which can combine with lipid peroxide to reduce ROS, so as to play an important role in antioxidant. The body’s lipid antioxidant system is regulated by GPX4 as its principal regulator. To protect biofilm systems against ferroptosis damage, GSH is employed as a cofactor to convert peroxide (R-OOH) into alcohol (R-OH) and decrease the toxicity of lipid peroxides. However, the body’s decreased GSH levels displays impacts on GPX4 activity, which is required for ferroptosis to occur. Numerous synthesis routes, such as glutathione synthetase (GSS) and nicotinamide adenine dinucleotide phosphate, are the source of GSH (121). A disulfide bond connecting the heavy chain SLC3A2 and the light chain SLC7A11 creates the cystine-glutamate reverse transporter protein known as the XC-system. It mediates the 1:1 exchange of glutamate and cystine inside and outside the cell. The extracellular glutamate concentration influences the transport rate of the XC-system, and an elevated glutamate concentration inhibits cystine uptake and GSH production, which results in altering the GPX4 activity alteration and ferroptosis (11, 122).

This XC-system/GSH/GPX4 axis is one of the main pathways that HG induces ferroptosis. According to Zhao et al., system XC-mediated suppression of ATF3 activity resulted in the induction of osteoblast ferroptosis in high glucose conditions, and these occurrences aided in the pathogenesis of T2DOP (19). They found that ATF3 was upregulated by HG *in vivo* and *in vitro*, which reduced the expression of SLC7A11 and the amounts of intracellular GSH and extracellular glutamate (19). ATF3 inhibition then boosted GPX4 levels and decreased the buildup of ROS and lipid peroxides and these modifications reduced osteoblast ferroptosis and enhanced osteogenic activity. According to Ma et al., osteoblasts from osteoporotic individuals with T2DM developed a lot of ferroptosis lipid peroxides. The down-regulated expression of GPX4 and SLC7A11 in osteoblasts mitochondria and the XC-system were correlated with these lipid peroxides (9).

In summary, high glucose condition induces the imbalance of iron metabolism (ferroptosis and iron overload) via abundant pathways like Nrf2/HO-1, METTL3, XC- system/GSH/GPX4. Some proteins, such as METTL3 and DMT1, also contribute dramatically to the regulation of iron metabolism. It indicated the necessary to explore deeply on the association between iron and bone metabolism and underlying pathways.

## 5. Iron-related signaling pathways and bone formation

### 5.1. NRF2/HO-1/GPX4

**Figure 2:**
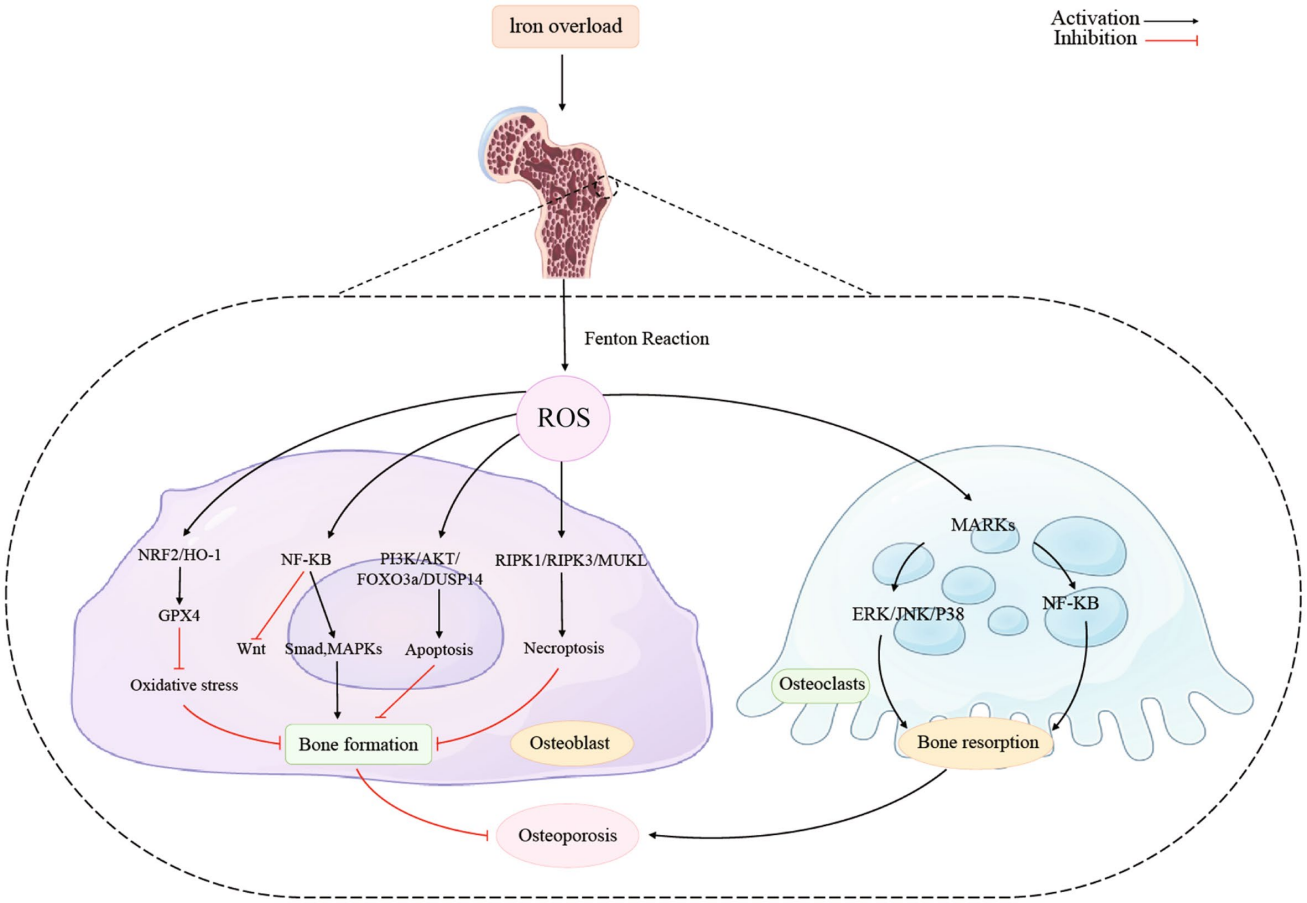
Iron overload and osteoporosis in osteoblast and osteoclast.

Figure 2 showed the association between iron overload and osteoporosis in osteoblast and osteoclast. Activating the NRF2/HO-1 channel considerably lowers ferritin levels while reducing oxidative stress and it prevents ferroptosis and promotes bone production (124). The nuclear factor erythroid 2-related factor 2 (Nrf2) signaling pathway is directly downstream of ROS and controls the transcription of antioxidant response element-dependent genes to sustain cellular redox homeostasis and regulate oxidative mediators (125). Recent studies demonstrated that melatonin activated the Nrf2/HO-1 pathway and increased levels of the antioxidant enzymes HO-1 and NAD(P)H dehydrogenase [quinone] 1 to prevent kidney damage caused by diabetes and exert neuroprotective effects (126, 127). Additionally, it has been noted that Nrf2 guarded cancer cells from ferroptosis brought on by erastin or RSL3 (128).

Researchers have found the NRF2/HO-1/GPX4 pathway had an impact on osteoblast. Ma et al. reported that activation the NRF2/HO-1 channel considerably lowers ferritin levels while reducing oxidative stress. NRF2 initiates the cellular peroxidation and defense process by activating the downstream enzymes glutathione peroxidase and superoxide dismutase (SOD). Additionally, it eliminates hazardous elements like ROS, and further reduce the toxic effects to osteoblasts (9, 129).

Furthermore, in ferroptosis, the antioxidant system Nrf-2/HO-1 could be suppressed. In the absence of Nrf-2, the activity and expression of the GPX4 protein is reduced and the severity of iron death is enhanced. It indicates that both the Nrf-2/HO-1 antioxidant system and iron death may be regulated under inflammatory settings (130). Additionally, researchers found the Nrf2/GPX4 pathway played an important role in age-related osteoporosis. Using 18 female wild type and 16 Nrf2-knockout (KO) mice as experimental subjects, Kubo et al. found that old Nrf2-specific KO mice showed reduced bone mass, which significantly implied that chronic Nrf2 deficiency made a great contribution to the progression of osteoporosis specifically in aging females (131). Yang et al. determined that 1,25(OH)2D3 can delay age-related osteoporosis via activating Nrf2 antioxidant signaling pathway and inhibition of oxidative stress, which provided support for the significant impact Nrf2 signaling pathway had on age-related osteoporosis (132, 133). Moreover, by evaluating the effect of 1,25(OH)2D3 on the Nrf2/GPX4 signaling pathway in MC3T3-E1 cells, other researchers also concluded that VDR activation inhibited osteoblast ferroptosis by activating the Nrf2/GPX4 signaling pathway, which indicates that there is a broad and profound link between the association of iron death and osteoblast (134).

### 5.2. NF-κB signaling pathways

To limit osteogenic development, nuclear factor κB (NF- κB) produces inflammatory molecules, suppresses Wnt signaling, and stimulates Smad and MAPK signaling pathways in osteoblasts. These changes caused by NF- κB mentioned above will ultimately activate ferroptosis (135, 136). Through its control over the production of a network of inducers and effectors that characterize responses to pathogens, NF-κB plays a crucial part in the cellular stress response as well as in inflammation (137). Inflammatory cytokines are released as a result of host defense mechanisms in reaction to inflammation, which activates the NF-κB pathway (138). Postnatal bone development requires BMPs, which also promote the expression of the matrix proteins osteocalcin and bone sialoprotein. Osteopenia, bone fragility, and spontaneous fracture are caused by a decrease in BMP activity (139, 140). The Wnt signaling system also promotes bone growth. When Wnt signaling is activated, β-nuclear catenin’s expression rises, which in turn causes osteocalcin and bone sialoprotein to express more strongly. Inflammation inhibits Wnt signaling by increasing the expression of Wnt antagonists such as Dkk1 or sclerostin (82).

NF-κB regulates transcription positively in practically every conditions. The latest research has shown that NF-κB may interfere with the transcription of gene and chemokines were suppressed when noncanonical NF-κB subunits bound to the κB sites (141, 142). Interferon-b expression at the degree of promoter is directly suppressed by the activation of noncanonical NF-κB (143). Thus, RelB-p52 heterodimers were formed because of the noncanonical pathway activation, which caused NF-κB to have a detrimental impact. In Tarapore’s study, the researchers discovered that NF-κB was crucial for the decreased production of matrix proteins brought on by inflammatory reactions, which eventually affected bone formation. Activation of NF-κB inhibits the production of matrix proteins both Wnt- and BMP-stimulated. This suppression entailed b-catenin and Runx2 inhibition by binding to neighboring consensus sites and NF-κB, to directly interacted with the involvement of response elements in the promoter regions of bone matrix proteins (144). Furthermore, Other studies also found that significant impacts of NF-κB on bone formation, by approving that it stimulates inflammatory factors and stimulates Smad and MAPK signaling pathways in osteoblasts to prevent osteogenic differentiation (107, 144, 145).

### 5.3. PI3K/AKT/FOXO3a/DUSP14

Iron overload significantly suppresses osteoblast proliferation and induces apoptosis through the PI3K/AKT/FOXO3a/DUSP14 channel, thus inhibiting bone formation in HG. It has been discovered that the PI3K/AKT signaling pathway contributes to signal transmission that is connected to cell proliferation, differentiation, invasion, and apoptosis (146). Specifically, researchers have reported that the proliferation and development of rat osteoblasts required activation of the PI3K/AKT signaling pathway (147).

The FOXO3a gene belonging to the FOXO subfamily. The transcription of FOXO3a is suppressed by pAKT, which regulates the phosphorylated process of FOXO3a. Members of the DUSP family are intimately connected to cellular proliferation as well. According to a prior study, DUSP4 promotes the growth and invasion of colorectal cancer cells. Xia et al. discovered that iron overload reduced osteoblasts growth and promoted apoptosis greatly through the PI3K/AKT/FOXO3a/DUSP14 channel (148). By noticing that the impact of iron overload in osteoblasts was greatly reduced by overexpressing DUSP14, their team demonstrated that through the inhibition of DUSP14 expression, iron overload may endanger the proliferation of osteoblasts. Additionally, iron overload enhanced p-AKT and p-FOXO3a expression in osteoblasts. FOXO3a could directly attach to the DUSP14 promoter and DUSP14 may therefore represent a unique element in the PI3K/AKT/FOXO3a pathway (149). In summary, PI3K/AKT/FOXO3a/DUSP14 signaling pathway is potentially in charge of cell defense in the presence of iron overload stress.

### 5.4. RIPK1/RIPK3/MLKL

In the iron overload-induced osteoblast apoptosis process, ROS could promote phosphorylation of RIPK1 and RIPK3 and create a positive vicious circle involving RIPK1/RIPK3/MLKL. Sufficient evidences suggest oxidative stress induced by iron overload is the primary factor in the pathophysiology of osteoporosis (150–152). It also appears that iron toxicity is intimately linked to cell death in illnesses from iron overload (153). Apoptosis and necrosis have been historically considered to be the two primary fundamental processes of cell death (154). ROS, as was already established, were crucial for the apoptosis that was induced by iron overload in the osteoblasts. Nevertheless, Tian’s research revealed that necrosis may also be strongly related to the characteristics of osteoblasts death from iron overload (155). Similar occurrences have been observed in earlier research, which indicated that necrosis may be the principal mechanism of cell death for osteoblastic cells in iron overload-associated bone disorders (156).

The precise mechanisms through which iron overload induces osteoblastic cells to necrotize remains not fully understood. An example of planned necrosis is necroptosis, which is distinguished by morphological variations of necrosis and is greatly reliant on regulating RIPK1, RIPK3, and MLKL. The phosphorylated MLKL eventually goes to the plasma membrane via oligomerization and penetrates, and then triggers necroptotic cell death (157, 158).

Tian’s team firstly demonstrated how ROS were crucially regulated in iron overload-induced necroptosis and found that ROS brought on by iron overload encourage necroptosis by creating a positive feedback loop with the involvement of RIPK1/RIPK3. The results of Tian’s study showed a dose-dependent rise in RIPK1 and RIPK3 phosphorylation as well as total protein expression in the osteoblastic cells following exposure to FAC. Nonetheless, following FAC treatment, the osteoblasts’ protein expression of MLKL showed no appreciable change. The addition of Nec-1, GSK872, or NSA inhibited iron overload-induced necrotic cell death in osteoblasts. Their findings illustrated iron overload induced necroptosis in osteoblasts cells, at least partially through the RIPK1/RIPK3/MLKL pathway, and finally inhibited bone formation (155).

In summary, iron absorption, storage, and excretion abnormalities, together with the aberrant expression of iron-related proteins IRP2, FtMt, TFR1, TFR2, HEP, and ferritin ferroxidase, may result in alterations in iron content. Multiple signaling pathways like NRF2/HO-1, PI3K/AKT/FOXO3a/DUSP14, RIPK1/RIPK3/MLKL, and NF-κB, are warranted be explored more for the targeted interventions of the imbalanced bone remodeling process.

## 6. Iron-related signaling pathways and bone resorption

Osteoclasts are multinucleated large cells that are differentiated from bone marrow monocytes and come from the hematopoietic cell lineage (64). Two essential cytokines, macrophage colony-stimulating factor (M-CSF) and receptor activator of nuclear factor-B ligand (RANKL), affects the development of monocytes into osteoblasts. The cytokine M-CSF regulates the process by which BMMSCs differentiate into preosteoblasts and their proliferation, whereas RANKL controls the process by which preosteoblasts differentiate into osteoblasts and the activity of mature osteoblasts (159). Furthermore, cytokines such as tumor necrosis factor (TNF) and interleukin (IL) (160) could regulate the formation of osteoblasts (161). It was shown that RANKL was also linked to the recruitment of the non-receptor tyrosine kinase and tumor necrosis factor-associated receptor (TNFR) (162). c-Src acts to activate signaling pathways involved in osteoclast differentiation and maturation, such as NF-κB signaling pathway (163), and MAPK signaling pathway while TNFR acts to activate the Akt signaling pathway, which in turn induces the expression of nuclear factor of activated T-cell (NFATc). NFATc is the core transcription factor of osteoclasts, which ultimately mediates osteoclast differentiation, fusion and degradation of inorganic and organic bone matrix (164). The common signaling pathways for osteoblasts include OPG/RANKL/RANK, NF-κB, c-src-PIK3-AKT, MAPK, and CN-NFAT, all of which were approved crucial for controlling osteoclast development (165). However, the latest research revealed that the NF-κB signaling pathway and the MAPK signaling pathway were mostly responsible for T2DOP in the case of ferroptosis caused by HG conditions (20).

### 6.1. NF-κB signaling pathway

The intrinsic immune system’s NOD, LRR, and pyrin domain-containing protein 3 (NLRP3) inflammatory vesicles recognize pathogens like viruses and bacteria, and activate inflammatory factors to mediate inflammation. It has been discovered that in osteoclasts, however, NLRP3 played a critical role in promoting osteoclast maturation and increasing bone resorption (166). A recent study showed that mice osteoclasts that expressed NLRP3 in particular did not undergo systemic inflammation. The amount of osteoclasts stayed the same, but the bone mass decreased by around 50% (165). The NLRP3 inflammasome performs a variety of tasks in both young and old persons. Bone loss in old mice lacking NLRP3 is increased through bone resorption rather than bone formation. Similarly, MCC950 inhibited osteoclast development by reducing caspase-1 activation, but not observed in young mice. Moreover, the transcription factor NF-κB, could encourage the production of molecules that control the development of inflammatory vesicles with the NLRP3 gene (163). And it’s demonstrated that the ROS generated in the high glucose state led to the phosphorylation of MAPK-related proteins, which in turn activated the MAPKs pathway and subsequently the NF-κB pathway. This increased the expression of NLRP3 in the internal environment, which in turn promoted the maturation of osteoclasts and increased osteoclastic bone resorption (163).

### 6.2. ERK/JNK/p38 pathway

Three different signaling pathways of MAPK, MAPK kinase (MEK or MKK), and kinase of MAPK kinase (MEKK or MKKKK), make up the MAPK signaling system. Together, these three kinases that can be activated in any order, regulates a range of significant physiological and pathological reactions, including cellular development, differentiation, stress, and inflammatory responses (167). ERK, JNK, p38/MAPK, and ERK5 are the four primary branching points of the MAPK pathway. JNK and p38 have comparable roles in inflammation, apoptosis, and cell growth; and the ERK pathway primarily controls cell growth and differentiation; and Ras/Raf protein serves as its upstream signal. These kinases used in the branching route are all different and can be used as biomarkers in the pathway.

As a downstream branching pathway of the MAPK pathway, ERK/JNK/p38 pathway is another signaling pathway that might contribute to osteoporosis. Related studies have shown that the ERK/JNK/p38 pathway plays an important role in promoting the differentiation of preosteoclasts, promoting the survival of osteoclasts and inhibiting osteoclast apoptosis (164). In contrast, iron deficiency with moral hyperglycemia enhances the expression of ROS increases, which in turn increases the expression of RANKL, thus promoting the ERK/JNK/p38 pathway for greater differentiation of pro-osteoclasts (164). This increases the bone resorption effect of osteoclasts, causing a disruption in the homeostasis of bone resorption and bone formation, which in turn leads to osteoporosis.

## 7. Therapeutic targets and drugs targeting iron metabolism for DOP

### 7.1. Preclinical monitoring: evaluating diabetes-specific risk factors for osteoporosis

In addition to the age-related risk factors and other established fracture causes, a comprehensive investigation of risk variables is required for the clinical examination of bone fragility in diabetes patients. Bone fragility is a distinct risk factor for fractures in both T1DM (168) and T2DM (169), and is substantially linked with the length of the condition. Individuals with T1DM are more likely to fracture more frequently and experience bone loss even when they are young (131, 170). Due to the fact that osteoporosis is a frequent complication of T1DM, DXA testing, and laboratory checks to identify additional risk factors, such as hypogonadism would be generally recommended by physicians. Hofbauer et al. advised testing for blood 25-hydroxyvitamin D (25[OH]D) in diabetics who were institutionalized (i.e., Living in a care facility such as a nursing home) or at risk of falls and fractures in order to identify a rapidly curable cause of falls and fractures. The initial bone assessment for determining fracture risk would also strongly be taken into consideration in the testing.

Poor glycaemia control was identified strongly associated with increased bone fragility, with a HbA1c threshold of more than 9% (75 mmol/mol) in individuals with T2DM and more than 9% (63 mmol/mol) in individuals with T1DM (171). Moreover, routine assessments should be made of hypoglycemic episodes, which can result in cardiovascular events, falls, and fractures in both type 1 (172) and type 2 (173) of diabetes. Consequently, it is advised to maintain stringent glycemic control in individuals who are younger and have the condition earlier. Strict glycaemia control’s skeletal benefits in patients with long-term disease, diabetic comorbidities, and a history of falls must be weighed against the elevated risk of falls and cardiovascular events brought on by hypoglycemia. Currently, sulfonylureas and thiazolidinediones are used cautiously in patients at risk of fractures, metformin, glucagon-like peptide-1 (GLP-1) receptor agonists, SGLT2 inhibitors, and DPP-4 inhibitors exhibits a safe bone profile for type 2 diabetes (174, 175). Moreover, Metformin was found to lower the incidence of fractures in T2D patients and increased bone mass and bone quality in ovariectomized (OVX) rats. The underlying mechanism contained decreased RANKL expression and osteoclast inhibition (176, 177). Another study demonstrated that metformin limits bone marrow stromal stem cells’ ability to produce succinate and lessens the stimulatory effects of succinate in promoting osteoclast development, and bone resorption (178); while a recent study reported that metformin usage did not increase BMD (179), and similar osteo-protective effect was also seen in non-diabetic OVX (180). Further studies are needed for the inconsistent findings in clinical practice.

### 7.2. General interventions and classic anti-osteoporosis drugs

This is a consensus that unless their serum 25(OH)D concentration is at least 20 ng/ml, all diabetics are recommended to take vitamin D supplements. In obese patients, calorie restriction to lower body weight is frequently used to halt the onset of diabetes mellitus; nevertheless, weight loss is considered to be linked to decreased bone mass. Thus, it is strongly advised that people with T2DM and obesity control their weight by carefully supervised exercise (181), which could strengthen bones aid patients with diabetes mellitus in preventing bone loss. According to a meta-analysis, people with T2DM who follow a Mediterranean diet rich in fresh fruits, vegetables, and fish have a lower incidence of fractures and microvascular sequelae (182). The unhealthy eating habits of high sugar and fat should be quitted. Also, bad lifestyle choices like drinking too much alcohol and smoking need to be carefully avoided.

Some clinical trials illustrated that alendronate (183) and teriparatide (184) displayed some therapeutic effects in diabetes mellitus through post-hoc analyses. According to Langdahl’s study, teriparatide showed similar effects in lowering fracture risk for diabetic patients as general patients (185). Alendronate has been shown to reduce postmenopausal osteoporosis patients’ fasting glucose and insulin resistance in preclinical diabetes mellitus (186). Dagdelen et al. found that alendronate had a more muted effect on increasing forearm BMD in postmenopausal osteoporosis patients with diabetes mellitus than in postmenopausal osteoporosis patients without diabetes mellitus, but it had no appreciable difference in effect on BMD in the hip and vertebrae between the two patient groups (187). Some studies proved that the mechanism of postmenopausal osteoporosis is also related to iron metabolism. For example, the postmenopausal spine may be protected against bone loss by dietary iron (188). Ni et al. suggested and tried to testify that an alternative method of treating postmenopausal osteoporosis might be to induce ferroptosis in osteoclasts by inhibiting Hypoxia-Inducible Factors (HIF-1) and ferritin (189). A recently created anti-osteoporosis medication called Romosozumab, the first sclerostin inhibitor licensed by the U.S. FDA, targets sclerostin, has demonstrated remarkable effectiveness in treating postmenopausal osteoporosis (190). Now that Picicca et al. demonstrated that diabetes caused osteocytes to alter over time and upregulate the sclerostin gene, we assumed that Romosozumab may be a very effective drug in treating DOP reasonably, which is also a potential research direction (85).

### 7.3. Pharmacological regulating iron metabolism and anti-ferroptosis therapies for DOP

**Figure 3:**
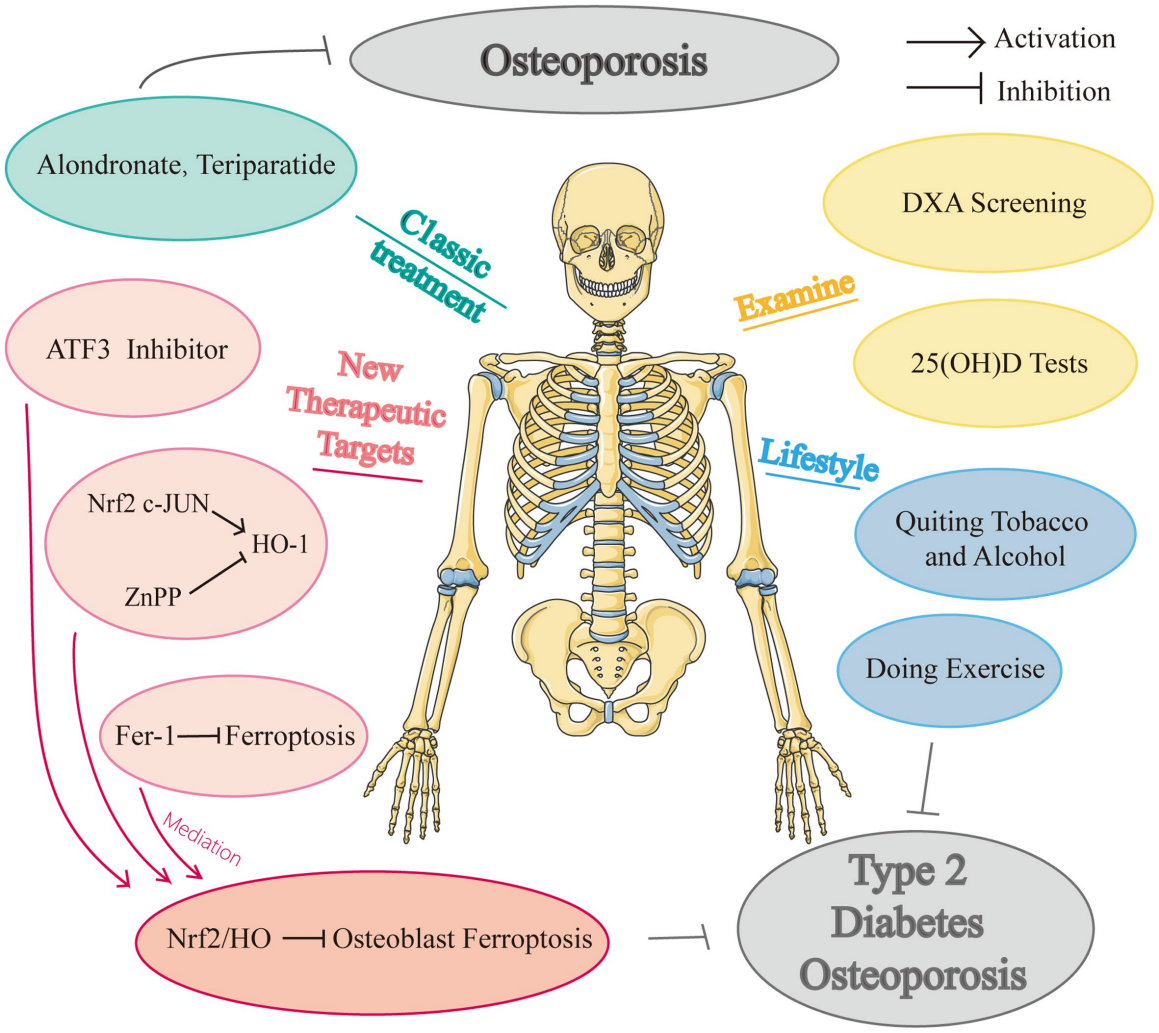
Drugs and potential therapeutic targets about iron metabolism in high glucose condition.

To the best of our knowledge, there remain no randomized controlled trials examining the effectiveness and security of anti-ferroptosis medications in individuals with diabetes osteoporosis. And there are no specific medications to treat DOP currently, and many studies merely explored the potential treatment impact in animal tests, with many studies focusing on simply the prospective therapeutic targets. We know that the distance from animal studies to clinical trials is long and this would be a potential research direction, people are all looking forward to a potent drug for DOP.  Figure 3 showed the drugs and potential therapeutic targets about iron metabolism in high glucose condition.

Iron affects several phosphate and bone illnesses, as was previously indicated (191). Iron homeostasis should always be maintained for healthy cellular activity. Many studies have revealed that the major feature of ferroptosis is iron excess-induced aberrant iron metabolism. Increased iron intake decreased stable iron, and iron outflow would together induce ferroptosis. The six-transmembrane prostate epithelial antigen 3 (STEAP3) transforms ferric iron to ferrous iron when the Tfr1 on the cell membrane binds to circulating iron. DMT1 then releases divalent iron into the cytoplasm’s labile iron pool (LIP). Notably, because of their significant LIP storage, lysosomes are considered to be the major organelles responsible for cellular ferroptosis, which shows potentially desirable potential disease targets (192).

Moreover, iron overload-induced liver ferroptosis in transferrin knockout mice is greatly reduced by both treatments with Fer-1 and hepatocyte-specific Slc39a14 deletion (193). Deferoxamine, an iron chelator, inhibits ferroptosis, has been demonstrated its clinical potential. Bordbar et al. found that in comparison to other regimens, combination therapy with Deferasirox and Deferoxamine had the greatest effect on lowering blood ferritin, despite its negligible value, and decreasing bone loss in the lumbar spine and femoral neck (194). Accordingly, Fer-1, was found to be an effective ferroptosis inhibitor because of its ability to scavenge lipid (195). Emerging studies have indicated that ferroptosis is involved in metabolic disease, cardiomyopathy, neurodegeneration, ischemia–reperfusion injury, and the effects of cancer (89, 196). Targeting ferroptosis may be an effective strategy for treating DOP.

Yang et al. applied a mouse model of DOP and established the critical involvement of ferroptosis in DOP-induced osteocyte death both *in vivo* and *in vitro* (10). The increased expression of HO-1 caused intracellular iron overload and heme breakdown, which subsequently triggered the oxidation of lipids. For this mechanism to work, nuclear factor-like 2 and c-direct JUN’s binding were required. Furthermore, inhibiting ferroptosis greatly reversed trabecular degeneration and osteoclast death. Iron atrophylinkage and HO-1 activation are causally connected and may result in a self-feeding vicious cycle. These all offered prospective therapeutic targets for upcoming DOP therapy plans: ZnPP (an HO-1 inhibitor) and Fer-1. Intriguingly, treatment with Fer-1 consistently had a higher therapeutic outcome than that with ZnPP, indicating that using Fer-1 to scavenge intracellular lipid peroxides may be a more effective treatment plan for DOP. Furthermore, their study showed ZPP and Fer-1 therapy in diabetic mice also prevented lacunar emptying and osteocyte death in addition to restoring trabecular balance. In conclusion, stopping the ferroptosis pathway could prevent DOP and osteocyte mortality in diabetic mice.

In addition, System Xc, an amino acid antiporter that is made up of two subunits of the xCT light chain (catalytic subunit, encoded by the SLC7A11 gene), and the heavy chain (chaperone subunit, encoded by the SLC3A2 (197, 198)), mediates the exchange of extracellular cystine and intracellular glutamate on the cell membrane. The expression level of SLC7A11 is typically positively correlated with the activity of the antiporter, playing a critical role in preventing ferroptosis caused by lipids. Because the light chain encoded by SLC7A11 is responsible for the primary transport activity, and the heavy chain subunit SLC3A2 primarily serves as a chaperone protein (19). Several research have demonstrated the therapeutic benefits of melatonin, which is a strong endogenous antioxidant. Thus, if melatonin may neutralize ROS, could this be a possible method by which melatonin treats DOP? Ma’s study might provide the solution (9). It has been demonstrated that melatonin can enhance bone microstructure both *in vivo* and *in vitro* by inhibiting osteoblasts’ ability to ferroptosis in which melatonin lowered ROS levels, elevated SLC7A11 levels, and boosted GPX4 activity by opening the NRF-2/HO-1 antioxidant channel Also, it reduced the toxicity of lipid peroxides to shield the biofilm system from ferroptosis, enhancing osteoblast ‘s capacity for osteogenesis and bone microstructure (9). Another study discovered that melatonin can inhibit the ERK signaling pathway and lower osteoblast autophagy levels, delaying the pathological development of DOP (199).

In other disease-related osteoporosis, such as postmenopausal osteoporosis, there are some specific medicines or therapeutic schedules. For example, some researchers suggested that some special types of osteoporosis, anti-resorptive medication should be used after anabolic therapy, similar to the therapeutic sequence used to treat common osteoporosis (200). And the best BMD improvements were seen in postmenopausal women with osteoporosis who received these sequential medications in this order in clinical studies (201). However, this has not yet been established for those with diabetes, and there is no clinical trial to prove this therapy (190). But this can be a potentially effective treatment option. According to Zhang et al. (93), postmenopausal osteoporosis is prevented by hepcidin-induced reductions in iron concentration and PGC-1 expression, which adversely affect osteoclast differentiation, so maybe hepcidin can play a role in treating DOP in the future?

## 8. Conclusion and outlooks

Long-term, poorly controlled diabetes commonly culminates in diabetic bone disease with fragility fractures, which has a considerable impact on socioeconomic and public health burdens. The advent of knowledge of the biological mechanisms and implicated pathways, coupled with improved multiscale imaging of bone, has made it feasible to gain new insights into the increased bone fragility in diabetes at many levels. In this review, we systematically summarize the diverse mechanism and pathways of ferroptosis in osteoblasts, osteoclasts, and other key cells, and attempt to comprehend the regulatory targets of interventions and treatments in clinical practice, applying the identified biomarkers as guides, aiming to highlight the near-term opportunities to elaborate the execution mechanisms and targeted therapeutics of iron metabolism and ferroptosis to T2DOP. For further research, it is necessary to clarify the diagnostic criteria for DOP in patients of varying ages and disease trajectories, and to reach a consensus. Although there have been some studies exploring the mechanism of iron metabolism and ferroptosis in diabetic bone loss, the mutual effect among these key proteins and pathways remains unclear, and the relative importance of each mechanism in the development of diabetic osteoporosis has not been explored, which is meaningful to find key therapeutic targets. Finally, there is no specific medicine to treat diabetic patient with osteoporosis, therefore developing new treatment strategy for patients with DOP is promising and significant. Advances in iron metabolism and ferroptosis are particularly noteworthy.

## Author contributions

HY, ZZhao, and JB contributed to conception and design of the review. JB, YY, DZ, ZZhuo, TS, HL, and ZH performed the literature search, drafted the manuscript, and constructed the figures and tables. HY and ZZhao reviewed the manuscript. All authors contributed to the article and approved the submitted version.

## Funding

This work was supported by National Natural Science Foundation of China (81571022), Multi-center clinical research project of Shanghai Jiao Tong University School of Medicine (DLY201808), and Shenzhen Science and Technology Funding (JCYJ20220530142205013).

## Conflict of interest

The authors declare that the research was conducted in the absence of any commercial or financial relationships that could be construed as a potential conflict of interest.

## Publisher’s note

All claims expressed in this article are solely those of the authors and do not necessarily represent those of their affiliated organizations, or those of the publisher, the editors and the reviewers. Any product that may be evaluated in this article, or claim that may be made by its manufacturer, is not guaranteed or endorsed by the publisher.
